# Successful combination of endoscopic and laparoscopic removal of multiple ingested needles: A case report

**DOI:** 10.1097/MD.0000000000019343

**Published:** 2020-02-21

**Authors:** Kota Tsuruya, Osamu Chino, Yoichi Tanaka, Yoshimasa Shimma, Shingo Tsuda, Masahiro Kikuchi, Hirokazu Shiozawa, Jun Aoki, Tomoki Nakamura, Tomoko Hanashi, Takayoshi Suzuki, Masashi Matsushima

**Affiliations:** a Department of Internal Medicine, Digestive and Liver Disease Center, Tokai University Tokyo Hospital, Tokyo; b Department of Internal Medicine (Gastroenterology), Tokai University School of Medicine, Isehara; c Department of Surgery, Digestive and Liver Disease Center, Tokai University Tokyo Hospital; d Department of Gastroenterology, National Hospital Organization Tokyo Medical Center, Tokyo, Japan.

**Keywords:** endoscopy, foreign body, laparoscopy, sharply pointed object, treatment plan

## Abstract

**Rationale:**

Foreign body (FB) ingestion is a relatively common clinical situation in the emergency department. However, multiple sharply pointed foreign bodies located in different organs are rare conditions and no definite treatment guidelines has been established.

**Patient concerns:**

A 31-year-old amateur magician visited the outpatient clinic with a chief complaint of epigastric discomfort. He might have accidentally swallowed some needles while practicing a magic trick 2 days before.

**Diagnosis:**

Imaging tests revealed 1 needle was stuck in the left liver lobe through the stomach wall, 1 was in the third portion of the duodenum, 3 were in the ascending colon, and 2 were in the transverse colon.

**Interventions:**

A needle in the duodenum and 5 in the colon were removed by endoscopy. The needle stuck in the liver from the stomach was not visible inside the stomach and was successfully removed by laparoscopy a few days later.

**Outcomes:**

The patient was able to tolerate an oral diet and was discharged on postoperative day 4 without any complications.

**Lessons:**

Developing a treatment plan in cases of multiple sharp FB may be difficult. A multidisciplinary team of endoscopists and surgeons is needed to determine the best possible treatment plan. This experience illustrates the importance of the planning of the sequence and method of removal of multiple foreign bodies from the gastrointestinal tract.

## Abstract 2

Supplemental Digital Content is available in the text

## 1 Introduction

Foreign body (FB) ingestion is not a rare condition treated by gastrointestinal endoscopists.^[1]^ Generally, the principles of management and treatment strategy of FB ingestion is indicated after some diagnostic evaluation, and sharp pointed FB detected in the esophagus, stomach, or duodenum require urgent endoscopic removal.^[2]^ However, a few cases involving multiple sharply pointed FBs in different digestive organs have been reported. Treatment plans may be difficult to formulate for FBs located in multiple organs. This case report highlights the endoscopic removal of multiple sewing needles located in the duodenum and colon and the laparoscopic removal of one that had migrated into the left liver lobe.

## 2 Case report

**Figure 1:**
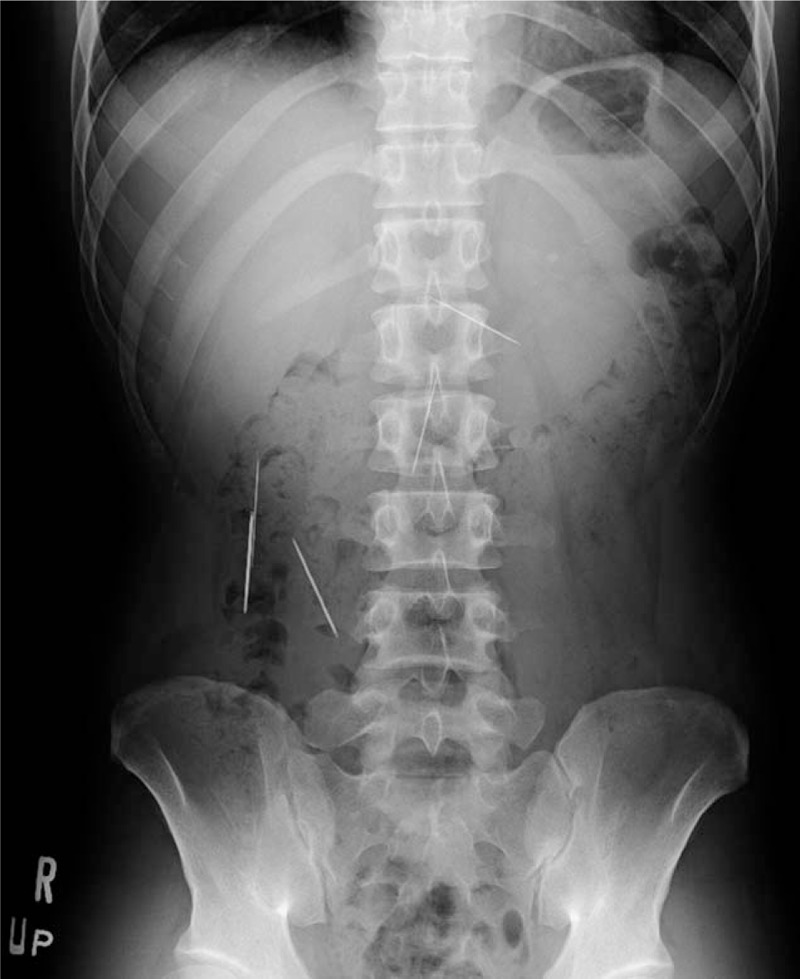
Abdominal radiograph showed multiple sewing needles in the abdominal cavity.

**Figure 2:**
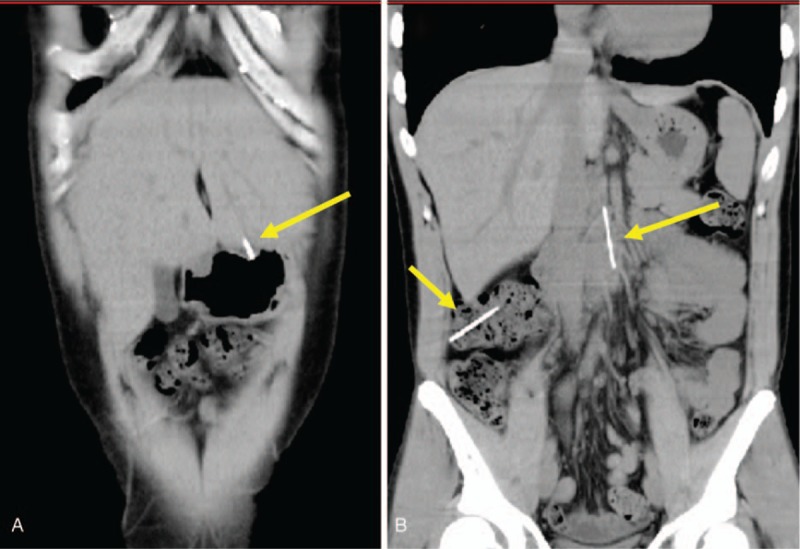
Computed tomography of the abdomen. (A) One needle was stuck in the left liver lobe through the stomach wall (yellow arrow). (B) Other needles were located in the third portion of the duodenum and transverse colon (yellow arrows).

**Figure 3:**
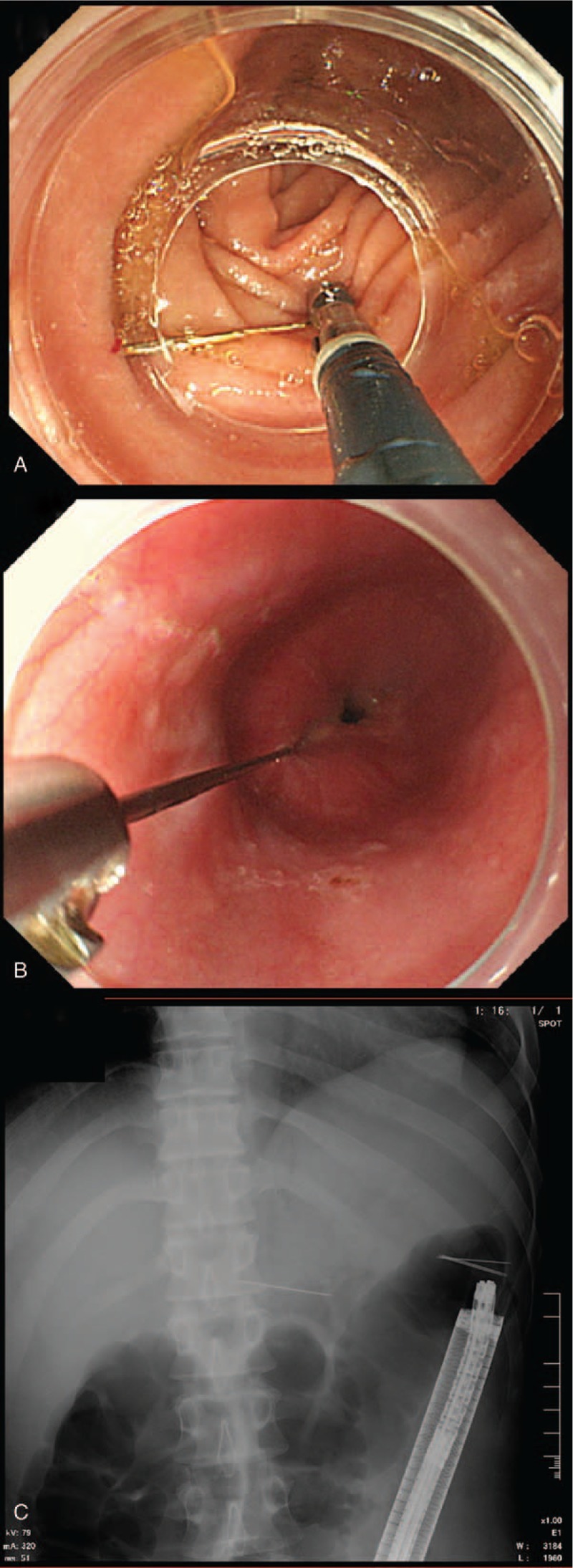
Endoscopic removal procedures for the 6 needles in the gastrointestinal tract. (A) Pediatric colonoscopy with a transparent cap was used while pulling the needle in the third portion of the duodenum back with the grasping forceps. (B) After grasping the needle tip using the forceps, the needle was drawn into the cap of the endoscope and removed with the overtube. (C) The needles in the colon were removed by a lower gastrointestinal endoscope through a sliding tube without complications.

**Figure 4:**
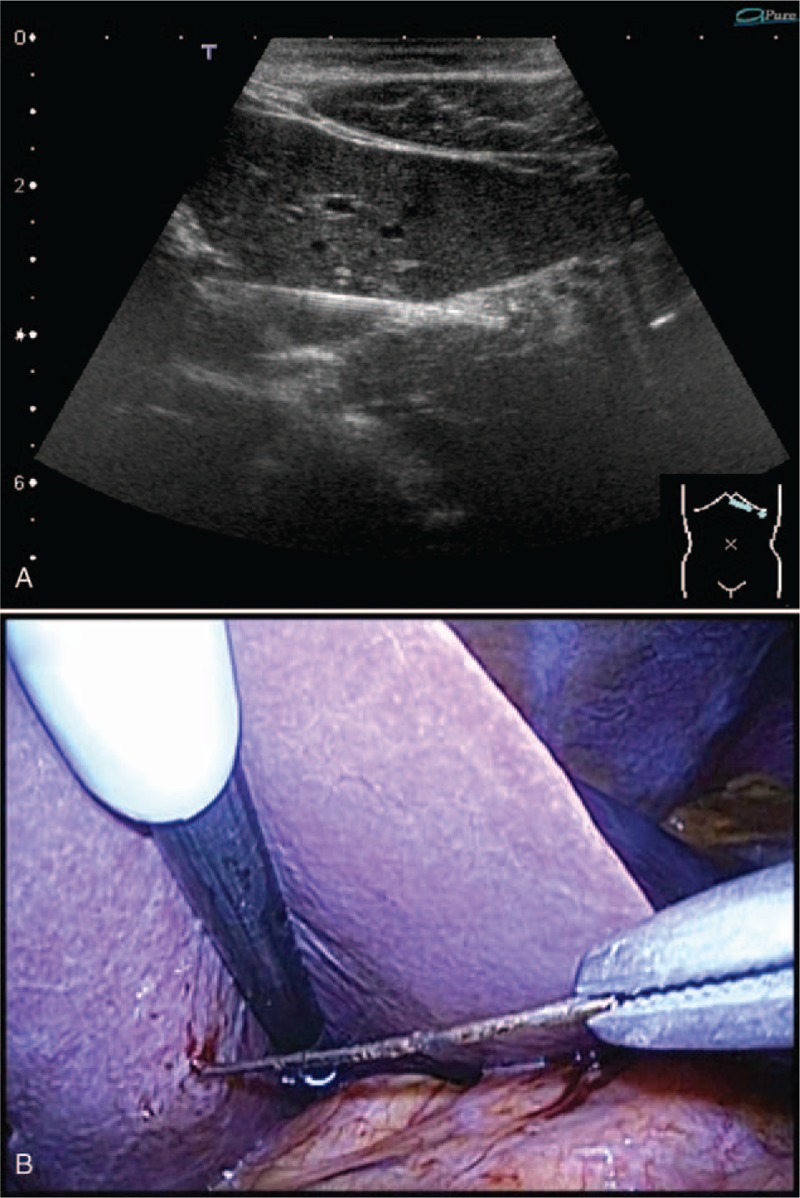
(A) Abdominal ultrasonography demonstrated that the needle stuck in the liver was gradually advancing toward the left portal vein. (B) The needle that had migrated into the left liver was successfully removed by laparoscopic surgery without any complications.

A 31-year-old man amateur Japanese magician visited the outpatient clinic of the hospital with a chief complaint of epigastric discomfort. He stated that he might have accidentally swallowed some needles while practicing a magic trick 2 days before. His medical, social, and family histories and physical examination findings were unremarkable. Laboratory tests revealed no abnormal values. An abdominal radiograph demonstrated multiple needles in the abdominal cavity (Fig. 1). Further examination by computed tomography (CT) revealed their exact locations: 1 needle was stuck in the left liver lobe through the stomach wall (Fig. 2A), 1 was in the third portion of the duodenum (Fig. 2B), 3 were in the ascending colon, and 2 were in the transverse colon (Fig. 2B). No free air or ascites was present in his abdominal cavity. The endoscopists and surgeons concluded that an endoscopic approach would be the initial step. First, we tried to remove a needle in the duodenum. A pediatric colonoscope with a transparent cap was inserted into the third portion of the duodenum. The needle that had become partially inserted into the duodenal wall was pulled back to the stomach using a grasping forceps (FG-7L-1; Olympus, Tokyo, Japan) (Fig. 3A) and was temporarily released in the stomach. After insertion of the overtube (MD-48518; Sumitomo Bakelite, Tokyo, Japan) to the esophagus, the needle was then entrained into the overtube by a conventional upper gastrointestinal endoscope with the forceps and removed with the overtube (Fig. 3B). The needle stuck into the liver through the stomach wall was not visible from the inside of the stomach, suggesting its complete impaction and migration into the liver. Next, we tried to remove the needles in the colon. After preparation for colonoscopy, the 5 needles in the colon were gathered together in the transverse colon near the splenic flexure. The needles in the colon were removed by a conventional colonoscope and the forceps through a sliding tube (ST-C3; Olympus) (Fig. 3C). Thus, 6 needles were successfully removed by upper and lower gastrointestinal endoscopic procedures without complications; however, the one needle stuck in the liver could not be removed by an endoscopic procedure. The needle had gradually advanced more deeply into the liver getting close to the left portal vein as shown by abdominal ultrasonography (Fig. 4A). Therefore, the last needle was successfully removed by laparoscopy a few days later (Fig. 4B, Supplementary Video1). As a result, the 7 needles in the duodenum, colon, and liver were all successfully removed by a combination of endoscopic and laparoscopic procedures without any complications. The patient was able to tolerate an oral diet and was discharged on postoperative day 4. The patient has no complication including liver abscess at 6-month follow-up.

## 3 Discussion

FB ingestions are relatively common in daily endoscopic practice. Approximately 80% to 90% of ingested FB pass through the intestinal tract without intervention, while 10% to 20% can be handled by endoscopic procedures, and approximately 1% require surgery.^[3]^ Large, thin, and sharp objects have a high risk of penetration of the digestive tract, and the risk of a complication such as mucosal ulceration, abscess, peritonitis, or fistula formation caused by a sharply pointed object is reportedly as high as 35%.^[4]^ Hence, sharply pointed objects detected in the esophagus, stomach, or duodenum require urgent endoscopic removal.^[5]^ The choice of appropriate retrieval devices, such as retrieval graspers, grasping forceps, baskets, snares, and nets, is important in the endoscopic retrieval procedure.^[2]^ When retrieving ingested sharply pointed objects, use of a transparent cap or rubber hood at the front of the endoscope and proper gripping devices to draw the sharply pointed objects out while keeping them in the endoscopic view reduce the risk of procedure-related perforation or mucosal injury.

The present case involved 7 very sharp needles in different parts of the digestive tract; thus, removal of the needles required endoscopy and/or surgery. The endoscopists and surgeons collectively decided that endoscopy would be the initial approach for the following 2 reasons. First, endoscopy is less invasive than surgery. In this instance, large laparotomy incision would have been required if surgery had been chosen, because the needles were distributed in different digestive organs. Second, bowel preparation was necessary to identify the exact location of the needles and to keep a clean operation field even if surgery were chosen as the initial approach. In other words, the condition after bowel preparation would also be ideal for an endoscopic approach.

Bowel perforation or penetration by ingested FB has been reported in all parts of the gastrointestinal tract; however, the duodenum and ileocecal and rectosigmoid regions are especially high-risk parts because of angulation or narrowing. In a report on toothpick ingestion, the injury sites were distributed among the duodenum (25%), sigmoid colon (14%), ileum (9%), jejunum (7%), cecum (7%), ascending to descending colon (7%), and rectum (7%).^[6]^ In the present case, the initial targets were the 2 needles in the stomach and duodenum because the former actually penetrated into the liver and the latter was located very near to the abdominal aorta.

For needles that have migrated into the liver, laparotomy or laparoscopic procedures are mostly used for removal.^[7]^ In some stable cases such as a lack of symptoms or immobilization of the needle, a wait-and-see approach may result in a benign course. Such no treatment cases were asymptomatic with normal laboratory data and were kept under observation over the years.^[8–10]^ Whereas, a FB in the liver that has become inserted into a vessel is associated with a risk of bloodstream infection or thrombus formation.^[11]^ In the present case, the needle gradually advanced more deeply into the liver and closely approached the left portal vein. We, therefore, attempted to remove the migrated needle by laparoscopy and succeeded in withdrawal of the needle without any complications. If all needles had been removed by laparoscopy or laparotomy, surgical time might have been much longer in addition to cut and suitable damage on duodenum and colon at least. Since endoscopic removals of the other needles were performed first, the remaining needle in the liver could be removed by a less invasive laparoscopic procedure in a shorter time compared with removing all needles with laparotomy or laparoscopy.

Clinical guidelines as well as various case reports have indicated the principles of management and treatment of ingested FB.^[2,3,5]^ However, the guidelines do not include complicated cases such as those involving multiple sharply pointed FBs located in different organs with a high risk of perforation or penetration, and the treatment plan was difficult to develop in the present case. Actually, the endoscopists and surgeons discussed and promptly decided on the treatment plan immediately after admission of the patient. The first target was the needle in the third portion of the duodenum very close to the abdominal aorta; this needle was successfully removed endoscopically. In contrast, the second target needle that advanced from the stomach wall into the liver could not be observed from inside the stomach after removal of the first needle. The head of the needle barely seemed to remain inside the stomach according to the CT scan before the admission, as depicted in Fig. 2A. Whether it remained inside the stomach before the first endoscopic trial is unknown, but a more flexible plan such as determining the first target after endoscopic observation of the stomach might be another choice.

We succeeded in removing multiple sharply pointed objects by a combination of endoscopic and laparoscopic procedures without complications. Developing a treatment plan in such cases may be difficult. Endoscopists and surgeons should therefore engage in prompt discussion and determine the best possible treatment plan cooperatively. This experience illustrates the importance of the correct combination and order of several FB removal procedures.

## Acknowledgment

The authors thank Angela Morben, DVM, ELS, from Edanz Group, for editing a draft of this manuscript. Their patient has provided informed consent for publication of this case.

## Author contributions

**Conceptualization:** Kota Tsuruya, Shingo Tsuda.

**Data curation:** Kota Tsuruya, Osamu Chino, Yoichi Tanaka, Yoshimasa Shimma, Shingo Tsuda, Masahiro Kikuchi, Hirokazu Shiozawa, Jun Aoki, Tomoki Nakamura, Tomoko Hanashi.

**Investigation:** Kota Tsuruya.

**Supervision:** Osamu Chino, Masashi Matsushima.

**Writing – original draft:** Kota Tsuruya.

**Writing – review & editing:** Osamu Chino, Yoichi Tanaka, Shingo Tsuda, Masahiro Kikuchi, Takayoshi Suzuki, Masashi Matsushima.

Kota Tsuruya orcid: 0000-0002-9884-8039.

## Supplementary Material

Supplemental Digital Content
